# Chronic Stress During Adolescence Impairs and Improves Learning and Memory in Adulthood

**DOI:** 10.3389/fnbeh.2015.00327

**Published:** 2015-12-11

**Authors:** Lauren E. Chaby, Sonia A. Cavigelli, Amy M. Hirrlinger, James Lim, Kendall M. Warg, Victoria A. Braithwaite

**Affiliations:** ^1^Center for Brain, Behavior, and Cognition, Pennsylvania State UniversityUniversity Park, PA, USA; ^2^Department of Ecosystem Science and Management, Pennsylvania State UniversityUniversity Park, PA, USA; ^3^Institute of the Neurosciences, The Huck Institutes of the Life Sciences, Pennsylvania State UniversityUniversity Park, PA, USA; ^4^Department of Biobehavioral Health, Pennsylvania State UniversityUniversity Park, PA, USA; ^5^Veterinary and Biomedical Sciences, Pennsylvania State UniversityUniversity Park, PA, USA; ^6^Department of Biology, Pennsylvania State UniversityUniversity Park, PA, USA

**Keywords:** adolescence, chronic unpredictable stress, learning, memory, reversal learning, *Rattus norvegicus*, laboratory rat

## Abstract

**HIGHLIGHTS**

- This study tested the effects of adolescent-stress on adult learning and memory.
- Adolescent-stressed rats had enhanced reversal learning compared to unstressed rats.
- Adolescent-stress exposure made working memory more vulnerable to disturbance.
- Adolescent-stress did not affect adult associative learning or reference memory.

Exposure to acute stress can cause a myriad of cognitive impairments, but whether negative experiences continue to hinder individual as they age is not as well understood. We determined how chronic unpredictable stress during adolescence affects multiple learning and memory processes in adulthood. Using male Sprague Dawley rats, we measured learning (both associative and reversal) and memory (both reference and working) starting 110 days after completion of an adolescent-stress treatment. We found that adolescent-stress affected adult cognitive abilities in a context-dependent way. Compared to rats reared without stress, adolescent-stressed rats exhibited enhanced reversal learning, an indicator of behavioral flexibility, but showed no change in associative learning and reference memory abilities. Working memory, which in humans is thought to underpin reasoning, mathematical skills, and reading comprehension, may be enhanced by exposure to adolescent-stress. However, when adolescent-stressed animals were tested after a novel disturbance, they exhibited a 5-fold decrease in working memory performance while unstressed rats continued to exhibit a linear learning curve. These results emphasize the capacity for stress during adolescence to transform the cognitive abilities of adult animals, even after stress exposure has ceased and animals have resided in safe environments for the majority of their lifespans.

## Introduction

During adolescence, mammals are remarkably sensitive to their environment and can undergo changes in behavior, physiology, and cognition that persist into adulthood (Romeo et al., 2006; Toledo-Rodriguez and Sandi, 2011; McCormick et al., 2012; Caruso et al., 2014; reviewed in Brown and Spencer, 2013; Green and McCormick, 2013). Why animals undergo this phase of plasticity remains unclear, but modifications during this transitional period may facilitate colonization and acclimation to new environments (Crone and Dahl, 2012). Important development changes in biological systems controlling reproduction, cognition, and the ability to respond to adversity typically occur during adolescence (Tanner, 1962; Spear, 2000; Romeo and McEwen, 2006). Stress can disrupt these developmental trajectories, and can cause lasting phenotypic alterations that may impact fitness, including reduced motivation for social interactions (Green et al., 2013) and exacerbated age-related cognitive decline (Sterlemann et al., 2010). Yet it appears that adolescent-stress can also enhance adult foraging-related problem solving abilities under threat (Chaby et al., 2015) and cause longer lasting threat associations compared with unstressed animals (Toledo-Rodriguez and Sandi, 2007), which could be advantageous in a dangerous environment. Given that experiences during adolescence can influence adult cognition, determining how exposure to stress during adolescence affects learning and memory processes remains a key goal for understanding developmental plasticity and the potential for developmental stress to shape life outcomes.

Adolescents may be more sensitive to stress for at least three reasons (*sensu* Romeo, 2013, 2015); (1) adolescents produce higher levels of glucocorticoid “stress” hormones in response to aversive physical and psychological stimuli compared with adults (McCormick et al., 2005; Romeo, 2010), (2) adolescents may be more sensitive to the effects of glucocorticoids on gene regulation (Lee et al., 2003), and (3) adolescent brain areas involved in stress regulation, learning, and memory (e.g., prefrontal cortex (PFC), hippocampus, and amygdala) are still developing and maturing during adolescence (Spear, 2000; Dahl, 2004). Brain structures integral in learning and memory processes, including the PFC and the hippocampus, undergo numerous maturational processes during adolescence that include the pruning and loss of large numbers of glutamatergic cells and increases in white matter density (Insel et al., 1990; Scherf et al., 2006; Jolles et al., 2011). It is suggested that stress may alter the maturation of these structures and affect their functioning later in life (Spear, 2000).

Changes in cognitive ability might affect fitness in at least two ways. First, cognitive ability can be a target of mate selection, and thereby affect reproductive output (Keagy et al., 2009; Verzijden et al., 2012). Second, increased cognitive abilities, such as learning and memory, can allow animals to maximize resource use in changing and complex environments (Papaj and Prokopy, 1988; Papaj and Vet, 1990; Dukas and Duan, 2000). For example, the ability to form an association between a predictive stimulus and a reinforcer (i.e., associative learning) is well-conserved across taxa and can facilitate exploitation of an environment by decreasing time spent searching for resources or by enhancing prediction and avoidance of threat (Dukas and Duan, 2000; De Houwer, 2014). Animals can cope with changes in the environment through reversal learning—i.e., abandoning previously established associations for alternative cues that were not previously reinforced (Clark et al., 2004). Reversal learning can be impaired shortly after stress exposure in adult rats (Cerqueira et al., 2007). Working memory, defined as holding information in memory for temporary use or manipulation (Hitch, 2002), is thought to constrain cognitive abilities including reasoning, reading comprehension, and mathematical skills, and is a more accurate predictor of academic success than IQ (Hitch and Baddeley, 1976; Carretti et al., 2009; Alloway and Alloway, 2010; Alloway and Passolunghi, 2011). Exposure to stress can impair working memory (Diamond et al., 1996, 1999) and deficits in working memory can reduce quality of life (Alptekin et al., 2005). Thus, determining if stressful experiences during adolescence can have long-lasting effects on adult learning and memory is important to understand the effects of developmental stress on fitness and well-being.

Stress exposure in adulthood can affect learning and memory processes (Luine et al., 1994; Kirschbaum et al., 1996). For example, the effects of stress can accrue over time and impair reference memory—i.e., the ability to retrieve information after a delay (Lupien et al., 2009; Nadel and Hardt, 2011). In adult rats, chronic stress can reduce spatial reference memory in an 8-arm water maze, but reference memory can recover after a 3 week delay in the absence of stress (Hoffman et al., 2011). However, chronic stress during adolescence (28–56 days of age) does not have an immediate effect on reference memory in the open Morris water maze, but impairs reference memory after a 3 week delay in the absence of stress (Isgor et al., 2004). Given that the developmental stage at stress exposure can shape the timing of responses to stress, it is important to assess these responses after a delay. Effects of exposure to stress in adolescence on behavior have repeatedly been detected from 25 to 196 days after stress exposure has ceased (Vidal et al., 2007; McCormick et al., 2008; Green et al., 2013; Chaby et al., 2014), but the duration of effects on cognitive processes are less well understood. Evidence suggests that these effects could be long lasting; in rats, exposure to daily isolation from weaning (21 days of age) to early adolescence (34 days of age) can impair reversal learning 28 days after stress exposure has ceased (Han et al., 2011) and spatial recognition memory can be impaired up to 12 months after social stress in adolescence (Sterlemann et al., 2010). Here, we examined whether stress in adolescence can affect learning and memory processes in late adulthood—after a longer delay than previously reported for these cognitive processes—but consistent with the timing of behavioral changes previously induced by the adolescent-stress paradigm used here (Chaby et al., 2015). To do this, we exposed adolescent rats to aversive unpredictable social, physical, and predation stimuli from 30 to 70 days of age. Rats were then housed without any manipulations but with basic enrichment for 106 days. After this delay, a battery of radial maze tasks were conducted to assess associative learning, reversal learning, working memory, and reference memory. We predicted that associative learning, a simple form of learning, would not be affected by adolescent-stress but that reversal learning, which may require behavioral flexibility (Bond et al., 2007), would be impaired. Similarly, we predicted that working memory and reference memory would be decreased by exposure to adolescent-stress.

## Methods

### Subjects and housing

**Figure 1:**
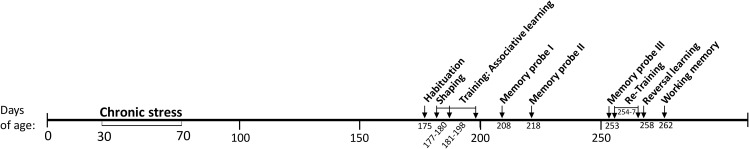
**Timeline of adolescent-stress manipulations and experiments**.

Male Sprague-Dawley rats (24) were obtained at 21 days of age from Harlan Laboratory (Frederick, Maryland). Animals were pair-housed in plastic cages, 20 × 26 × 45 cm, according to the National Institute of Health (NIH) recommendations described in the *Guide for the Care and Use of Laboratory Animals*. Basic enrichment items were added to all cages at 23 days of age (two 7.6 cm diameter PVC tubes hanging from the wire cage lid and two 2.5 × 2.5 × 8 cm pine blocks). All cages were kepts at 20–21°C and 40–45% relative humidity and cleaned weekly. Enrichment items were changed when soiled. Rats were kept on a 12:12 reversed light:dark cycle to accommodate testing during the dark phase when rats are most active; all testing began at least 2 h after the start of the dark phase and was completed within 6 h. Standard rat chow (LabDiet® 5001, 23% protein) and tap water were available *ad libitum* except preceding rewarded tests; food was removed 2 h before all rewarded tests to increase motivation for food rewards. A timeline of manipulations is given in Figure 1. To minimize disturbance the experimenter was not in the room during testing and experiments were video-recorded. Test chambers were sprayed with 70% ethanol solution and wiped clean between trials. Experiments were approved by the Pennsylvania State University IACUC, protocol #44459.

### Chronic unpredictable stress

Pair-housed rat cages were randomly assigned to the adolescent-stress treatment (*n* = 12) or the unstressed control group (*n* = 12). Each week between 30 and 70 days of age adolescent-stress rats encountered six stressors, three between 000 and 1200 h and three between 1200 and 2400 h. The three stressor types (physical, social, and predation) and order of stressor presentation varied, but were balanced so that each type of stressor was represented twice per week. This stress paradigm has previously induced long-term behavioral and cognitive changes and is described in more detail in Table 1 and Chaby et al. (2015). To account for handling and cage changes during the stressors, rats in the unstressed group were handled and transferred to clean cages approximately twice per week, coinciding with stressors that required a new cage. All rats were weighed weekly during the stress treatment, and every second week thereafter to monitor health. The duration of the stress treatment (30–70 days of age) included a short post-pubertal period in early adulthood (55–70 days of age) to cover the entire ontogenetic window of adolescence (Schmidt et al., 2007; Sterlemann et al., 2010) and to evaluate behaviors mediated by the prefrontal cortex, which continues to develop into early adulthood (Spear, 2000).

**Table 1:** **Chronic unpredictable stressor descriptions**.

	**Physical stressors**	**Duration**
Smaller cage	Rat pairs were housed in a cage 25% smaller than their home cage (Doyle et al., 2011).	4 h
Damp bedding	Rat pairs were housed with 200 ml of water mixed into 2/3 of the bedding of the home cage (Harding et al., 2004).	6 h
Cage tilt	Home cages were tilted at a 30° angle (Harding et al., 2004).	6 h
**SOCIAL STRESSORS**		
Isolation	Rats were housed individually in a clean cage with a 7.6 cm diameter PVC tube and a 2.5 × 2.5 × 8 cm pine wood block (McCormick et al., 2008).	1 h
Crowding	Sets of 2 rat pairs were combined into one clean cage (20 × 45 cm; Harding et al., 2004; Doyle et al., 2011).	4 h
Foreign bedding	Rat pairs were housed in a cage previously occupied by a pair of older conspecifics (Harding et al., 2004).	12 h
**PREDATION STRESSORS**		
Taxidermied bobcat	An adult male taxidermied bobcat was placed on a wheeled cart and moved continuously in front of the rat cages (varied from 1 to 6 feet distance; Blumstein et al., 2004).	30 min
Fox urine	Tink's Red Fox-P® was sprayed onto cotton balls enclosed in mesh and placed into the home cage (Fendt and Endres, 2008).	30 min
Cat fur	*Felis catus* fur was placed into the home cages, inside of mesh (Kendig et al., 2011).	30 min
Feline vocalizations	Bobcat, mountain lion, domestic cat, lion, and tiger territorial and aggressive vocalizations were played ~5 feet outside of the home cage (Chaby et al., 2015).	30 min

#### Radial maze: habituation

At 176 days of age, rats were placed in the center of the radial maze individually and allowed to explore for 5 min to familiarize rats with the testing environment (depicted in Supplementary Figure 1). We quantified entries of the radial arms as an indicator of baseline activity in the testing conditions. An arm entry was defined as crossing all 4 feet into an arm.

#### Radial maze: associative learning shaping

Rats underwent two shaping sessions prior to associative learning trials in order to familiarize them with consuming rewards in a single arm in the maze (the “correct” rewarded arm for the associative learning experiment). During shaping, a Cheerio® reward was placed halfway down one of the five arms of the radial maze. The rewarded arm was counterbalanced across treatment. In the first shaping session, rats were placed in the center of the maze and allowed to explore freely. In the second shaping session, rats began in the start chamber for three trials separated by inter-trial intervals of 30 s. Rats were separated into two groups of 12, balanced by treatment. One group of 12 underwent shaping trials at 177 and 179 days of age, the second at 178 and 180. During all shaping trials, rats were removed 1 min after consuming the Cheerio or after 5 min had elapsed.

### Associative learning

From 181 to 198 days of age rats underwent 12 days of associative learning trials (with 6 days of rest). Conditions of the associative learning trials were similar to the shaping trials; rats were given 3 trials per day with 30 s inter-trial intervals, and were removed 1 min after consuming the reward or after 5 min. Rats began each trial in the start chamber, but could not enter the radial maze until 20 s had elapsed and an opaque plastic barrier was removed via pulley. To control for visual cues, Cheerio rewards were located in a dish recessed into the maze floor so that rewards were not visible until a rat was standing over the reward dish at the end of the arm. To control for olfactory cues, Cheerios were placed alongside the arms on the outside of the maze. To assess associative learning, we recorded latency to enter the radial maze (all 4 feet inside radial maze), latency to find the food reward (rat's head dipped inside rewarded dish), whether a trial was “correct” (rewarded arm entered first), reference memory errors (enter an unrewarded arm), and working memory errors (re-enter an unrewarded arm in the same trial).

### Long-term reference memory

Rats underwent long-term reference memory probes at 10, 20, and 55 days after the last training day. Memory probes consisted of a single trial identical to associative learning trials. We recorded latency to enter the radial maze, latency to find the food reward, arms entries, and reference and working memory errors.

### Retraining and reversal learning

Rats were tested for reversal learning at 258 or 259 days of age. For the reversal learning test, and all subsequent parts of the experiment, rats were tested in two groups of 12, balanced by treatment and tested on alternate days. Prior to the reversal learning test, rats underwent 2 days of retraining (identical to the associative learning trials). After retraining, reversal learning was tested by moving the reward to a previously unrewarded arm and measuring latency to enter the radial maze, latency to find the food reward in the novel arm, and number of arms entered during each of two reversal trials (De Bruin et al., 1994). Conditions in reversal trials were identical to re-training, except for the position of the reward. We conducted a second reversal trial to determine whether behavior was consistent across both trials, indicating a potential motivational difference.

### Working memory and novel disturbance

The effects of stress exposure in adolescence on working memory and the ability to maintain a working memory after a novel disturbance in adulthood were tested at 261 and 262 days of age. Rats were exposed to a novel reward arm, distinct from the arms used in the associative and reversal learning experiments, over the course of three trials to create a working memory of a new reward location (Kesner, 2000; Cerqueira et al., 2007). These trials were identical to the associative learning trials except for the novel reward location. Following the third working memory trial, rats were exposed to a novel environment for 20 min to disrupt memory for the new reward location (Diamond et al., 1996). The novel environment was a circular gray plastic chamber (diameter 29 cm, height 36 cm) which had been wiped with a citrus orange cleaner. Citrus scents can be aversive (Amiri et al., 1998) and exacerbate stress responses in laboratory rats (Komori et al., 2003). Rats were then returned to the radial maze for two additional trials with the reward remaining in the same location as the earlier working memory trials. To determine whether any effects of the chamber could be explained by changes in activity, each arm was divided into quadrants and the number of crosses between quadrants was measured from video recordings.

### Consummatory extinction

At 314 days of age, motivation for a reward was tested, as this could mediate behavior in reward-based learning and memory tasks. We used a modified successive negative contrast test where animals are first familiarized with a reward, then the reward is made inaccessible and the degree of persistence to obtain the absent reward is quantified (Flaherty et al., 1979; Chaby et al., 2013). In the first phase of the test, rats were given access to a 32% sucrose solution for 5 min each day over 9 days in an opaque, plastic chamber (30.5 cm^3^). In the second phase of the test, rats were given 5 min in the same chamber for two additional days, but the solution was made inaccessible by a layer of plastic at the seam of the spout that was not visible when the bottle was positioned for the test. An open bottle of sucrose solution outside the opaque testing chamber provided olfactory cues similar to the reinforcing cues present in the first phase of testing. To quantify persistence we used an electronic device that registered each time a rat contacted the metal spout to obtain the reward (see Flaherty et al., 1979; Chaby et al., 2013).

### Data analysis

To determine the amount of time spent engaged in each learning and memory trial, latency to enter the maze was subtracted from the total latency to locate the reward for all trials. To conform to the assumptions for parametric analyses, we used a natural log transformation was used for latency to locate the reward and activity difference scores. To determine whether adolescent-stress exposure affected the number of arm entries during habituation to the radial maze, we used a two-tailed *t*-test. The effect of adolescent-stress on associative learning was tested by averaging the latency to locate the reward across the three trials per day, and using a repeated-measures general linear model (RMGLM) with stress condition and time as fixed factors. To assess associative learning we also tested the total number of correct trials each day with a RMGLM with stress condition and time as fixed factors. The effect of adolescent-stress on reference memory was tested in the three memory probes (each consisting of a single trial), using the latency to locate the reward and the number of errors in separate RMGLMs with stress condition and time as fixed factors.

Performance just prior to the reversal trials was tested using latency to locate the reward and number of arm entries in the three trials on the last day of re-training, using RMGLMs with stress condition and time as fixed factors. Number of arm entries was used because five adolescent-stressed and six unstressed rats made no errors by the completion of retraining. For the reversal learning trials, because only the first reversal trial was novel, measures from the first and second reversal trials were analyzed using separate GLMs with stress condition as a fixed factor. For the working memory trials were analyzed with RMGLMs with stress condition as fixed factors. For the working memory trials before the novel chamber, latency to locate the reward and the number of arm entries were analyzed with RMGLMs with stress condition and time as fixed factors. To assess whether the novel chamber induced changes in behavior (and to account for group differences in performance in the working memory trials prior to the novel chamber), we subtracted latency to locate the reward in the two trials after the novel chamber from the latency to locate the reward in last working memory trial. The same procedure was used for the number of arm entries and the activity measure. The resulting “difference scores” were analyzed with RMGLMs with stress condition and time as fixed factors. If a significant interaction between treatment and time was detected, we analyzed each time point individually with an analysis of variance (ANOVA). One rat from the adolescent-stressed group became distressed in the novel chamber and repeatedly attempted to jump out of the chamber. This rat was returned to his home cage, and was not included in re-exposure trials. No other rat exhibited signs of distress. To compare performance during the consummatory extinction test, we used a RMGLM with stress condition and time as fixed factors. Analyses were run in IBM® SPSS® Statistics v 21; values are reported as means ± standard error (SE). Statistical significance was assigned when *p* ≤ 0.05.

## Results

### Habituation

Adolescent-stress exposure did not affect the number of maze arm entries during the habituation task [stress average: 16.5 ± 0.8, unstressed average: 15.3 ± 0.9; *T*_(22)_ = 1.04, *P* = 0.31].

### Associative learning

**Figure 2:**
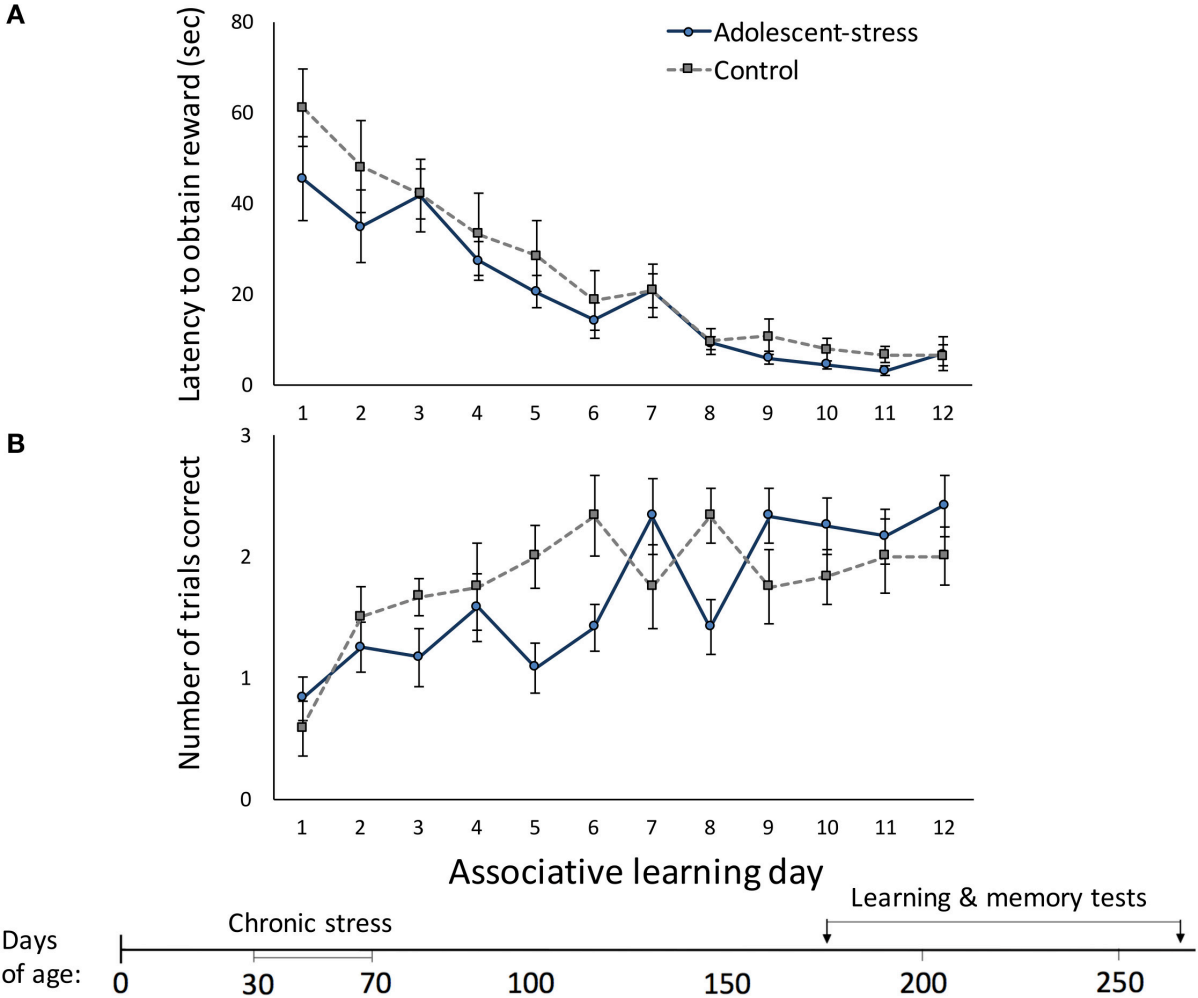
**The effect of stress during adolescence on associative learning in adulthood, measured by the latency to obtain the reward (A) and the number of correct trials out of three trials (B) in a radial arm maze, means ± standard error**.

Across the 12 trials, latency to located the reward decreased [*F*_(1, 22)_ = 96.94, *P* < 0.001] and number of correct trials per day increased [*F*_(1, 22)_ = 7.52, *P* < 0.001]. Stress during adolescence did not affect associative learning; there were no differences between adolescent-stressed and control animals in (a) latency to locate the reward [*F*_(1, 22)_ = 1.00, *P* = 0.33; Figure 2A] or (b) total number of correct trials each day [*F*_(1, 22)_ = 0.20, *P* = 0.66; Figure 2B]. Adolescent-stress did not affect the rate of improvement in the latency to locate the reward [stress × time interaction: *F*_(1, 22)_ = 0.70, *P* < 0.74; Figure 2A]. However, adolescent-stressed rats increased the number of correct trials over time more slowly than unstressed rats [stress × time interaction: *F*_(1, 22)_ = 3.63, *P* < 0.01; Figure 2B]. A *post-hoc* analysis revealed that rats exposed to stress in adolescence had fewer correct trials than unstressed rats on day 6 [*F*_(1, 22)_ = 5.04, *P* < 0.01; Figure 2B] and day 8 [*F*_(1, 22)_ = 5.04, *P* < 0.01; Figure 2B].

### Long-term reference memory

**Figure 3:**
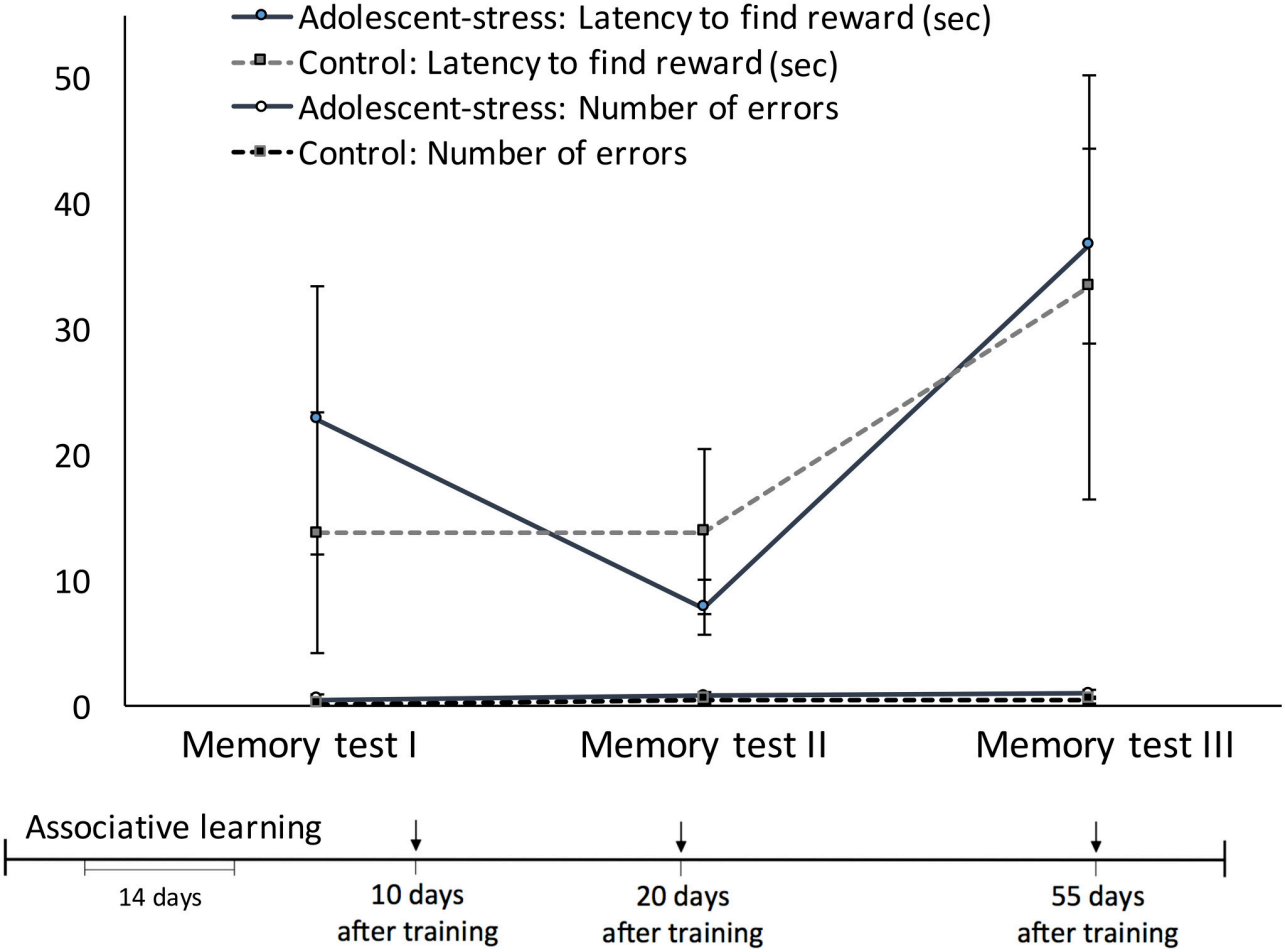
**The effect of chronic stress during adolescence on reference memory at three time points in adulthood, measured by the latency to obtain the reward and the number of entries into incorrect arms, means ± standard error**.

Across the 3 memory probes, starting 10 days after the associative learning trials, all rats exhibited an increase in latency to locate the reward over time [*F*_(1, 22)_ = 12.10, *P* < 0.00] but remained constant in number of errors [*F*_(1, 22)_ = 0.90, *P* = 0.41; Figure 3]. Adolescent-stress did not affect reference memory (Figure 3)—either latency to locate the reward [*F*_(1, 22)_ = 0.17, *P* = 0.69] or number of arm entry errors [*F*_(1, 22)_ = 1.25, *P* = 0.28]. On average, rats made less than one mistake in each of the three memory probes. Adolescent-stress did not affect the rate of change in reference memory (stress × time interaction) in either latency to locate the reward [*F*_(1, 22)_ = 0.30, *P* = 0.75] or the number of arms entered [*F*_(1, 22)_ = 0.04, *P* = 0.96].

### Re-training and reversal learning

Performance improved during retraining for all rats; latency to locate the reward [*F*_(1, 22)_ = 4.33, *P* = 0.02] and number of arm entries [*F*_(1, 22)_ = 6.37, *P* = 0.01] decreased over time. In the final re-training day, adolescent-stress did not affect latency to locate reward [*F*_(1, 22)_ = 0.12, *P* = 0.73; stress average: 11 ± 6 s, unstressed average: 13 ± 7 s] or number of arm entries [*F*_(1, 22)_ = 0.28, *P* = 0.61; stress average: 1.5 ± 0.2, unstressed average: 1.4 ± 0.2]. There were no stress × time interactions [latency to locate the reward, *F*_(1, 22)_ = 0.18, *P* = 0.84, number of arms entered, *F*_(1, 22)_ = 0.67, *P* = 0.52].

**Figure 4:**
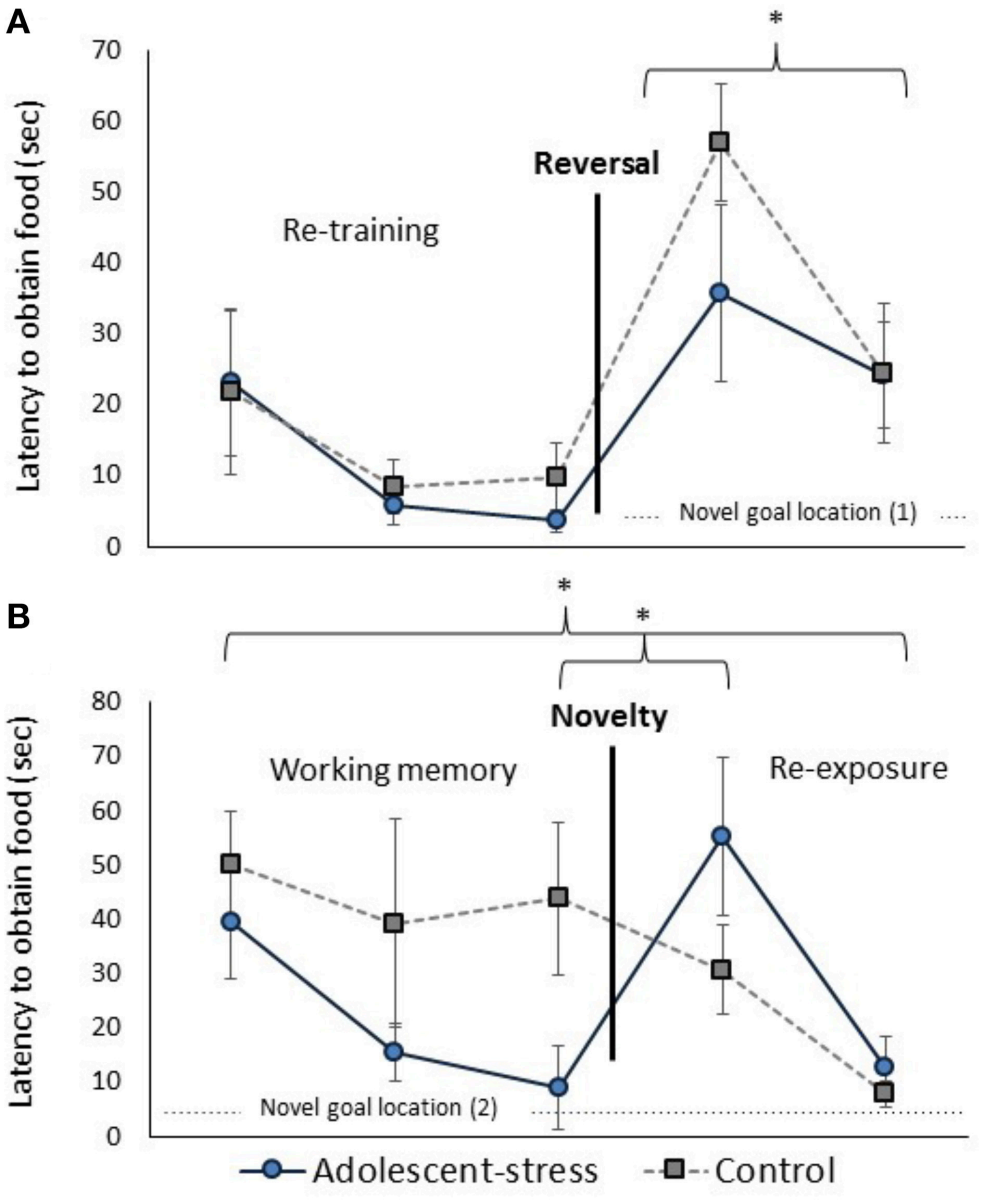
**The effect of stress during adolescence on reversal learning (A) and working memory (B) in adulthood, measured by the latency to obtain the reward**. Asterisks indicates *p* ≤ 0.05; means ± standard error.

Adolescent-stress enhanced reversal learning (Figure 4A); adolescent-stressed rats located the food reward 45% faster than unstressed rats in the first reversal trial [*F*_(1, 22)_ = 5.10, *P* = 0.04]. By the second reversal trial this effect had abated, after only a 30 s inter-trial interval, suggesting that motivation to obtain the reward was the same for both groups [*F*_(1, 22)_ = 0.02, *P* = 0.90]. This effect withstands a Bonferroni correction for multiple comparisons. The number of arm entries did not differ between groups in the first or second reversal trial [*F*_(1, 22)_ = 0.07, *P* = 0.79; *F*_(1, 22)_ = 0.40, *P* = 0.53].

### Working memory and novel disturbance

All rats exhibited a decreased latency to locate the reward across the working memory trials [*F*_(1, 22)_ = 9.40, *P* < 0.001; data in Supplementary Table 1]. Across the three working memory trials, before the novel chamber exposure, adolescent-stressed rats found the reward faster than unstressed rats [*F*_(1, 22)_ = 4.22, *P* = 0.05; Figure 4B]. The number of arm entries was not affected by adolescent-stress [*F*_(1, 22)_ = 0.86, *P* = 0.37; data in Supplementary Table 1] or time [*F*_(1, 22)_ = 2.19, *P* = 0.13; data in Supplementary Table 1]. After a Bonferroni correction for multiple comparisons, the difference in working memory loses statistical significance, though it is likely still a biologically relevant effect. There were no stress × time interactions for latency to find the reward [*F*_(1, 22)_ = 1.46, *P* = 0.24] or number of arm entries [*F*_(1, 22)_ = 2.23, *P* = 0.12].

In both re-exposure trials, adolescent-stress increased the effect of the novel chamber on performance [effect of stress: *F*_(1, 21)_ = 9.39, *P* < 0.01, stress × time: *F*_(1, 21)_ = 0.99, *P* = 0.32]. In the first trials after exposure to the novel chamber, adolescent-stressed rats showed a >500% increase in latency to find the reward while unstressed rats decreased their latency by 30%. In the second trial after the novel chamber, adolescent-stressed rats were still 5 ± 7 s slower to find the reward compared to their performance before the chamber, while the unstressed rats located the reward an average of 36 ± 15 s faster. This effect withstands a Bonferroni correction for multiple comparisons. Adolescent-stress did not affect number of arm entries in the re-exposure trials [*F*_(1, 21_ = 1.27, *P* = 0.27]. The difference in latency to locate the reward following exposure to the novel chamber was not explained by a change in activity [effect of stress: *F*_(1, 21)_ = 3.38, *P* = 0.10, stress × time: *F*_(1, 21)_ = 2.38, *P* = 0.16, data in Supplementary Table 2].

### Consummatory extinction

Stress during adolescence did not affect persistence to obtain a reward [*F*_(1, 22)_ = 0.45, *P* = 0.51]. Adolescent-stressed and unstressed rats exhibited a similar number of licks during the first trial with the inaccessible sucrose solution (stress average: 53 ± 7, unstressed average 47 ± 7) and during the second trial (adolescent-stressed: 48 ± 9, unstressed: 60 ± 13).

## Discussion

Exposure to chronic unpredictable stress during adolescence was found to shape adult cognition; adolescent-stress exposure had beneficial effects on some learning and memory processes, and detrimental effects on others, and no effect on yet other aspects of learning and memory in adulthood. Stress during adolescence did not affect associative learning or reference memory tested 4–6 months after adolescent-stress exposure. Despite this, adolescent-stressed animals showed enhanced reversal learning more than 6 months after stress exposure, compared to unstressed rats. Further, the ability to maintain a novel reward location in working memory in adulthood may be enhanced by adolescent-stress (though this effect was lost after a Bonferroni correction for multiple comparisons, it is likely still a biologically relevant effect). However, after a disruption (exposure to a novel chamber), adolescent-stressed rats increased their latency to locate the reward more than 5-fold. This decrease in performance was so strong that the performance of adolescent-stressed rats dropped to the level of the first working memory trial when they were naive to the reward location. Unstressed rats, however, continued to exhibit a linear learning curve even after the novel disturbance, suggesting a more robust working memory of the reward location. These changes in learning and memory could last the lifespan of *Rattus norvegicus*; the changes in working memory and reversal learning described here were detected shortly after the median lifespan of male Norway rats outside of captivity, ~250 days (Davis, 1948, 1953).

Exposure to the novel chamber, intended to disrupt working memory of the reward location, had opposite effects on performance in the two treatment groups; latency to locate the reward decreased by 30% in unstressed rats but increased more than 500% in adolescent-stressed rats. However, after all rats had been re-exposed to the reward location, in the second trial after the chamber (following only a 30 s inter-trial interval), adolescent-stressed rats matched unstressed rats in latency to locate the reward. This 500% change in performance over such a short delay between trials suggests that novelty-induced motivational differences cannot account for the increase in latency to locate the reward, but rather that adolescent-stress increases vulnerability to disturbance in working memory (Diamond et al., 1996). To further determine whether these were cognitive or motivational differences, we tested persistence to obtain a familiar reward made inaccessible in a consummatory extinction task (Flaherty, 1996; Cuenya et al., 2012). Our results showed that adolescent-stress exposure did not affect persistence for a reward, suggesting that reversal learning and working memory effects should not be attributed to motivational differences, but rather reflect changes in cognitive function. Similarly, activity could not account for performance differences because the adolescent-stress treatment did not affect either baseline activity or activity following the novel chamber. Furthermore, after exposure to the novel chamber, adolescent-stressed rats took longer to find the reward location but on average exhibited increased activity levels compared with their pre-chamber activity levels, indicating that inactivity does not explain the increase in vulnerability to disruption of working memory caused by adolescent-stress.

Our results highlight the importance of context when considering long-term effects of stress (e.g., Chaby et al., 2015). The importance of context is further demonstrated by the juxtaposition of our results with those described in Toledo-Rodriguez and Sandi (2007), which showed that adolescent-stress can enhance fear learning, a type of associative learning. In fear learning an innocuous stimulus is associated with an aversive stimulus, such as a shock or predator cue. Fear learning in adulthood can also be enhanced by isolation stress during early life (Lukkes et al., 2009). Animals exposed to early stress may have an advantage in fear learning assays, but not reward-based associative learning tasks, relative to unstressed animals, because the testing environment in fear learning tasks is more consistent with a stressful developmental environment, compared with reward-based learning environments (Love et al., 2005; Breuner, 2008; Sheriff and Love, 2013). Toledo-Rodriguez and Sandi (2007) also showed that exposure to stress in adolescence can inhibit extinction when a trained cue is presented repeatedly without the paired aversive stimulus; their results showed that when a cue that previously indicated a threat was made unreliable adolescent-stressed rats did not alter their behavioral stress response while unstressed rats attenuated their response. The differences in performance exhibited by adolescent-stressed animals in aversive vs. appetitive (reward-driven) learning environments suggest that environmental conditions in adulthood shape cognition, but also that rearing environment, and whether an adult testing environment is consistent with an animals early environment, also acts to shape cognitive processes.

Adolescence is characterized by heightened plasticity and behavioral flexibility (reviewed in Crone and Dahl, 2012). It is suggested that increased behavioral flexibility in adolescence may facilitate integration into novel social or environmental contexts following dispersal from natal environments (Crone and Dahl, 2012). Increased flexibility might also be advantageous in unpredictable, stressful environments later in life. It is possible that exposure to aversive or unstable conditions during adolescence could program an animal to maintain increased behavioral flexibility into adulthood, such as the enhanced reversal learning abilities demonstrated here. Heightened plasticity in adolescence is central to hypotheses that adolescence is a period of vulnerability and an opportunity for “programming” of future behavioral and physiological responses (reviewed in McCormick et al., 2010; Romeo, 2015). The capacity of adolescent-stress to have programming effects that persist throughout life is supported by lasting changes in the hypothalamic-pituitary-adrenal (HPA) axis caused by adverse experiences in adolescence; the HPA axis regulates the response to stress, including the production of glucocorticoid “stress” hormones, and can mediate persistent changes in behavior, such as those demonstrated here (Seckl, 2001; Pohl et al., 2007). Stress exposure in adolescence can affect glucocorticoid and mineralocorticoid mRNA expression and hippocampal size, which underpin a myriad of behavioral and cognitive processes (Isgor et al., 2004; Sterlemann et al., 2008). Global changes in glucocorticoid receptor expression and exposure to high levels of corticosterone during gestation can impair reversal learning in adolescence (Hayashi et al., 1998; Harris et al., 2013). The effects of adolescent-stress on the HPA axis can vary with sex, temperament, and the frequency of stressor presentation (Pohl et al., 2007; Schmidt et al., 2007; McCormick et al., 2008; Sterlemann et al., 2008; Caruso et al., 2014). It remains unclear why some models of adolescent-stress cause lasting changes in glucocorticoid production while others do not (e.g., Overmier and Murison, 1991; McCormick et al., 2005; Chaby et al., 2014; reviewed in McCormick et al., 2010), but these differences may be important for understanding the role of persistent changes resulting from stress. It is also important to consider when effects from stress in adolescence emerge; some effects may fade with time, while others may appear only after a delay. The predictive adaptive response hypothesis suggests that some effects of stress during early development manifest after a delay, and that these delayed effects have functions specific to an ontogenetic stage that can enhance fitness if the environment remains consistent with the early life environment (Gluckman et al., 2005). Indeed, delayed effects from stress exposure in early life have been documented, though more longitudinal studies are needed (Ganella et al., 2015). Following chronic stress in adolescence Isgor et al. (2004) found decreased hippocampal volume that appeared only after a delay. Similarly, effects from maternal separation can also appear after a delay, including decreases in both parvalbumin in prefrontal cortex interneurons and immunoreactivity to synaptophysin in the hippocampus (Andersen and Teicher, 2004; Brenhouse and Andersen, 2011). Further, developmental stage at the time of stress exposure can be important; stress exposure in adolescence (from 33 to 35 days of age) can have lasting effects on adult behavior that differ from the effects caused by stress exposure in juvenile development (from 27 to 29 days of age). Exposure to stress during both juvenile and adolescent development can impair avoidance learning, but learned helplessness is only affected by earlier juvenile-stress, and not by adolescent-stress (Tsoory and Richter-Levin, 2006).

Although it is difficult to predict how results from laboratory models might translate to free-living animals, it is important to note that without intervention the consequences of adolescent-stress can have lasting effects on behavior (Green et al., 2013), cognition (McCormick et al., 2012), and physiology (Isgor et al., 2004) that persist into adulthood. Following early life stress, however, exposure to environmental enrichment (e.g. toys, group housing) can reverse some lasting changes in behavior and physiology (Francis et al., 2002; Bredy et al., 2003, 2004; discussed in Romeo and McEwen, 2006). Although rescue effects from enrichment can be substantial, some effects of early stress persist even in enriched conditions, including changes related to learning (e.g., decreases in hippocampal long-term potentiation, Bredy et al., 2003) and future processing of stress [e.g., changes in corticotropin-releasing factor (CRF) gene expression, (Francis et al., 2002)]. In the current study, adolescent-stressed rats were housed with basic enrichment items and exposed to adverse unpredictable stimuli for 40 days. Following this, to assess the lasting effects of adolescent-stress, rats were housed in standard laboratory conditions with basic enrichment for 106 days. It is possible that in the absence of these basic enrichment items, the lasting effects from stress in adolescence could be even more severe. Contrastingly, in naturalistic environments, it is likely that animals are continually exposed to dynamic stimuli that can exacerbate or ameliorate the lasting effects of adversity in adolescence. Future studies are needed to determine how the effects of adolescent-stress on cognition manifest in naturalistic environments and how these effects might impact performance and fitness.

## Conclusions

Exposure to chronic unpredictable stress during adolescence was found to have both beneficial and detrimental effects on learning and memory processes in adulthood. Compared to rats reared without stress, adolescent-stressed rats exhibited enhanced reversal learning 188 days after stress exposure, suggesting adolescent-stress may increase behavioral flexibility in adulthood. Exposure to adolescent-stress also may enhance working memory 191 days later, which is suggested to underpin reasoning, mathematical skills, and reading comprehension in humans. However, working memory in adolescent-stressed animals was highly vulnerable to disturbance. The differences in working memory and reversal learning described here were seen shortly after the median lifespan of male Norway rats outside of captivity, ~250 days (Davis, 1948, 1953), suggesting that the influence of adolescent-stress on adult cognition can be life-long.

## Author contributions

LC designed the experiments and wrote the manuscript, LC, VB, and SC analyzed and interpreted the data and edited the manuscript. LC, AH, JL, KW, conducted and refined experimental procedures. All authors approved the final version to be published and agree to be accountable for all aspects of the work.

### Conflict of interest statement

The authors declare that the research was conducted in the absence of any commercial or financial relationships that could be construed as a potential conflict of interest.
