# Supplementary material for: Chronic Stress During Adolescence Impairs and Improves Learning and Memory in Adulthood
Source: Front Behav Neurosci. 2015 Dec 11;9:327. doi: 10.3389/fnbeh.2015.00327 (PMC4675857; doi:10.3389/fnbeh.2015.00327)
Supplement: Supplementary file 1 [file Table1.DOCX]

**Supplementary materials**

**S Table 1:** The effect stress in adolescence on arm entries in a working memory task.

|  | Adolescent-stressed rats: number of arms entered | Unstressed rats:  number of arms entered |
| --- | --- | --- |
| First working memory trial | 3.3 + 0.5 | 3.2 + 0.4 |
| Second working memory trial | 2.8 + 0.4 | 2.9 + 0.5 |
| Third working memory trial | 1.8 + 0.6 | 3.2 + 0.6 |

*****depicts means + standard error

**S Table 2:** The effect stress in adolescence on activity in a working memory task.

|  | Adolescent-stressed rats: activity (quadrants crossed) | Unstressed rats:  activity (quadrants crossed) |
| --- | --- | --- |
| Last trial before chamber | 15 + 5 | 24 + 5 |
| First trial after chamber | 21 + 3 | 16 + 3 |
| Second trial after chamber | 17 + 3 | 10 + 2 |

*****depicts means + standard error


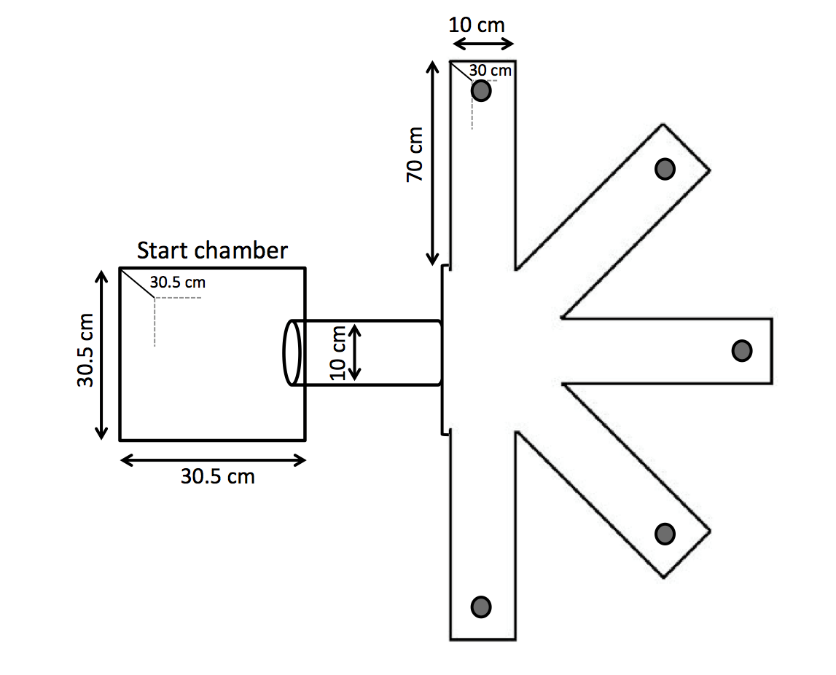


**S Fig. 1:** Radial arm maze schematic (not drawn to scale).
